# Tele-pharmacy perception, knowledge and associated factors among pharmacy students in northwest Ethiopia: an input for implementers

**DOI:** 10.1186/s12909-023-04111-9

**Published:** 2023-02-27

**Authors:** Masresha Derese Tegegne, Sisay Maru Wubante, Mequannent Sharew Melaku, Nebyu Demeke Mengiste, Ashenafi Fentahun, Wondwossen Zemene, Tirualem Zeleke, Agmasie Damtew Walle, Getnet Tadesse Lakew, Yonas Tsegaw Tareke, Mubarek Suleman Abdi, Hawariyat Mamuye Alemayehu, Eskedar Menkir Girma, Gizaw Getye Tilahun, Addisalem Workie Demsash, Hiwote Simane Dessie

**Affiliations:** 1 Department of Health Informatics, Institute of Public Health, College of Medicine and Health Sciences, University of Gondar, Gondar, Ethiopia; 2 Department of Health Informatics, Institute of Public Health, Mettu University, Mettu, Ethiopia; 3 Health Management Information System Unit, Gazo Woreda Health Office North Wello, Woldia, Ethiopia; 4 Health Management Information System Unit, Amhara Public Health Institution, Dessie, Ethiopia; 5 Health Management Information System Unit, Asossa Zonel Health Department, Benishangul-Gumuz, Ethiopia; 6 Health Management Information System Unit, Debark General Hospital, Debark, Ethiopia; 7 Health Management Information System Unit, Debremarkos Hospital, Debre Markos, Ethiopia; 8 Health Management Information System Unit, Enat Hospital, Debre Birhan, Ethiopia; 9 Departement of Health Informatics, School of Public Health, College of Medicine and Health Sciences, Wachemo University, Hossana, Ethiopia

**Keywords:** Ethiopia, Perception, Pharmacy students, Tele-pharmacy, Knowledge

## Abstract

**Background:**

Tele-pharmacy is a subset of telemedicine in which pharmacies use telecommunication technology to provide patient care. Tele-pharmacy can improve pharmaceutical care service delivery by reducing medication errors, improving access to health professionals and facilities in remote and rural areas, and minimizing adverse drug events. However, there is limited evidence regarding future pharmacists' knowledge and perceptions of the Tele-pharmacy system in Ethiopia. As a result, this study aimed to assess tele-Pharmacy perception, knowledge and associated factors among pharmacy students in Northwest Ethiopia.

**Methods:**

An institutional-based cross-sectional study was conducted among 376 pharmacy students in Northwest Ethiopia between July 15 and August 27, 2022. A pre-tested self-administered questionnaire was used to collect data. The data were entered using Epi info version 7.0 and analyzed using SPSS version 25. Descriptive statistics, bivariable and multivariable logistic regression analysis were used to describe pharmacy students' knowledge and perceptions of Tele-pharmacy and identify associated factors. An adjusted odds ratio (OR) and a p-value with a 95% confidence interval (CI) were calculated to declare statistical significance.

**Results:**

From a total of 352 participants, about 32.4% with [95% CI (27%-37%)] and 48.6% with [95% CI (43%—54%)] had good knowledge and a positive perception toward Tele-pharmacy, respectively. Being age group of 26–30 (AOR = 0.35, 95% CI: 0.17–0.68), being male (AOR = 2.38, 95% CI: 1.26–4.49), Having a CPGA of > 3.5 (AOR = 2.28, 95% CI: 1.24–4.19), Taking basic computer training (AOR = 2.00, 95% CI: 1.17–3.39), Management support (AOR = 1.84, 95% CI: 1.06–3.19) were found to be significantly associated with pharmacy students' knowledge of Tele-pharmacy. Similarly, having access to electronic devices (AOR = 3.80, 95% CI: 1.81–7.97), training related to pharmacy information systems (AOR = 6.66, 95% CI: 3.34–13.29), availability of guidelines (AOR = 2.99, 95% CI: 1.62–5.50) were found to be significantly associated with pharmacy students' perceptions of Tele-pharmacy.

**Conclusion:**

This study found that pharmacy students have limited knowledge and perceptions of the Tele-pharmacy system. A continuing Tele-pharmacy training package, incorporating pharmacy information system guidelines as part of their education, and providing managerial support could be recommended to improve pharmacy students' knowledge and perception of Tele-pharmacy.

## Introduction

Telemedicine refers to delivering health care and public education in rural and remote areas [1]. Telemedicine has grown steadily over the last decade as telecommunication technology has advanced and costs have decreased. Tele-pharmacy is a subset of telemedicine in which pharmacies use telecommunication technology to provide patient care [2]. Tele-pharmacy can potentially improve pharmaceutical care service delivery by reducing medication errors and adverse drug events [3]. Furthermore, Tele-pharmacy has the potential to benefit remote and rural areas with limited access to health professionals and facilities [4]. The practical and efficient use of health information technology will be of infinite importance; it will increase pharmacist accessibility, improve patient quality of life and satisfaction with healthcare services, minimize resources, and improve patient clinical outcomes [5].

Pharmacists today want to broaden their profession to provide more services to the rural community while also improving patient outcomes. As a result, Tele-pharmacy services such as medication orders, medication history reviews, dispensing drugs, remote patient consultation, therapeutic drug monitoring, and medication therapy management are becoming more common [6]. These services can be provided using eHealth tools like mobile consultation, software applications, and automated dispensing machines [7].

Pharmacists and student pharmacists should understand the application of telecommunication technology in the pharmacy field to provide the best services. Worldwide there is evidence showing that the proportion of pharmacy student knowledge of tele-pharmacy was 60.3% in the United States [8], 42% in Riyadh city of Saudi Arabia [9], and 67% in Malaysia [4]. Whereas the proportion of perception towards tele-pharmacy was 87% in the University of Tennessee [8], 70.6% in Jordan [10], 61% in Malaysia [4], and 40% in Pakistan [11]. Furthermore, evidences revealed that technological variables and socio-demographic characteristics were discovered to be the determinant factors associated to students' knowledge of and perceptions of the tele-pharmacy system [9–12].

Tele-pharmacy is one of the best options for providing community-based medication-related healthcare services, allowing pharmacists to address healthcare needs in developing countries like Ethiopia, where health professionals and healthcare facilities are scarce [13]. Furthermore, Tele-pharmacy was the best option for patients living in rural areas to reduce travel distance, save time, and access health care services, particularly for those over the age of 65 and those with disabilities [13, 14].

Ethiopian health sector transformation plan mentioned using health-related information technologies such as Tele-pharmacy as an essential transformation device to improve the quality of health care services [15]. Even though it is widely accepted that tele-pharmacy can help improve access and quality of healthcare delivery when distance is an issue, there is little evidence in Ethiopia about future pharmacists' knowledge and perceptions of tele-pharmacy.

The knowledge and perception of pharmacists students about Tele-pharmacy is a determining factor in the successful implementation of Tele-pharmacy services [16]. Since they play a vital role in the health care system and the functioning of Tele-pharmacy, investigating their knowledge and perception of tele-pharmacy is mandatory [17]. As a result, this study aimed to examine pharmacy students' knowledge and perception of tele-pharmacy.

The result of this study will provide baseline data for policymakers in solving the problem regarding the limited utilization of Tele-pharmacy and improving the knowledge and perception of pharmacy students. It will help them in planning an intervention based on the evidence generated by this study.

## Methods

### Study settings and design

The study was conducted among pharmacy students at the University of Gondar College of Medicine and Health Science, located in the historic town of Gondar, 726 kms northwest of Addis Abeba. The University of Gondar, formerly known as the Gondar College of Medical Sciences until 2003, is Ethiopia's oldest medical school, founded in 1954 as a Public Health College. It is located in northwest Ethiopia, 726 kms from Addis Ababa, Ethiopia's capital. According to data from the University of Gondar's college of medicine and health science school of pharmacy, 376 students were enrolled in their course. An institutional-based cross-sectional study was used to assess pharmacy students’ knowledge and perception of Tele-pharmacy and the associated factors.

### Study population and eligibility criteria

The study was carried out among pharmacy students at the University of Gondar College of Medicine and Health Science. The study included all pharmacy students enrolled in their courses at the University of Gondar College of Medicine and Health Science who were available during the data collection period. However, students who were ill and unable to complete the questionnaire were excluded from this study.

### Sample size determination and sampling procedure

There are currently 376 students enrolled in the pharmacy department at the University of Gondar College of Medicine and Health Sciences. The first-year students were not enrolled in the department at the time of data collection. This is because Ethiopia’s current ministry of health schedule requires all natural science students to attend a one-year common course before enrolling in their specific department. In the remaining years, students attended classes in the pharmacy department. There are 51 second-year students, 54 third-year students, 88 fourth-year students, 82 fifth-year students, and 101 post-basic and postgraduate students. Finally, all active pharmacy students in the pharmacy department at the University of Gondar College of Medicine and Health Science (*n* = 376) were invited to participate in this study.

### Study variables

The primary outcome variable of the study was perception and knowledge of Tele-pharmacy. The tools for this study were adapted from a review of related literature [4, 11, 18]. Some independent variables include socio-demographic and technological variables related to Tele-pharmacy perception and knowledge.

### Operational definitions

*Knowledge of Tele-pharmacy:* Ten items with "yes" or "no" responses were used to assess knowledge of Tele-pharmacy. For a total possible score of ten, each correct answer was worth one point, while each incorrect answer was worth zero points. A median of ten questions about Tele-pharmacy Knowledge was calculated. Those who scored higher than the median value were thought to have "Good knowledge about Tele-pharmacy," while those who scored a median value and lower were supposed to have "Poor knowledge about Tele-pharmacy" [4, 18].

*Perception towards Tele-pharmacy* was assessed using a 5-point Likert scale ranging from "strongly disagree" (score 1) to "strongly agree" (score 5). A median of 14 questions about Perception toward Tele-pharmacy was calculated. Those who scored higher than the median value were thought to have a "Good perception of Tele-pharmacy," while those who scored a median value and lower were supposed to have a "Poor perception of Tele-pharmacy” [4, 11, 18].

### Data collection procedure and quality control

A structured, pre-tested, and self-administered questionnaire was used to collect data. Six health information technology professionals collected the required data, And Two health informatics professionals with master's degrees and research experience oversaw the data collection. The principal investigators provided training for data collectors and supervisors two days before the start of data collection. Supervisors strictly supervised the data collection process and provided regular onsite advice and feedback to data collectors. The principal investigators and supervisor exchanged information face-to-face daily.

Before data collection, a pre-test was conducted on 10% of the sample size among pharmacy students at Bahirdar University. The questionnaire was checked for clarity, simplicity, understandability, completeness, consistency, and coherency during the pre-testing. Appropriate corrections were taken on time for completeness and accuracy before the beginning of data collection. The pre-test results were also used to assess the internal consistency of the questionnaire. Cronbach's alpha was used to determine the internal validity of the data collection instrument, and the scores on knowledge and perception of Tele-pharmacy were 0.86 and 0.98, respectively.

### Data processing and analysis

The collected data were entered into Epi info version 7.0 and transferred into SPSS version 25.0 software for further analysis. A table, graph, and text were used to present descriptive statistics. A bivariable logistic regression analysis was performed to determine each study variable's effect on the outcome variable. Variables with a p-value of 0.2 in the bivariate analysis would be entered into a multivariable logistic regression analysis to check for confounding effects on the bivariate analysis's association. The strength of the association would be determined using a 95% confidence interval odds ratio, and a p-value less than 0.05 would be considered a significant variable. The model was fitted with *p* = 0.34 according to homer's goodness of fit test, multi-collinearity was checked between independent variables, and all variance inflation factors were less than 3.

## Results

### Socio-demographic characteristics of participants

A total of 352 study subjects participated, with a response rate of 93.62%. The mean age of the study participants was 24.26 with an SD ± 3.419 years with ranges from 20 to 37 years. About 259 (73.6%) of the respondents were in the age category of 20–25. The majority, 274(77.8%) of the participants, were male, and 295 (83.8%) were single. Regarding religion, around 286 (81.3%) of the study participants were Orthodox, and 93 (26.4%) were post-basic and postgraduate students Table 1.

**Table 1:** Socio-demographic characteristics of pharmacy students

Variables	Category	Frequency	Percent
Age in years	20–25	259	73.6
	26–30	75	21.3
	> = 31	18	5.1
Sex	Male	274	77.8
	Female	78	22.2
Religion	Orthodox	286	81.3
	Muslim	42	11.9
	Protestant	24	6.8
Marital status	Single	295	83.8
	Married	40	11.4
	Divorced	17	4.8
Year of Study	2^nd^ year	47	13.4
	3^rd^ year	48	13.6
	4^th^ year	84	23.9
	5^th^ year	80	22.7
	Post basic and Postgraduate	93	26.4
Cumulative GPA	< 3.00	113	32.1
	3.0–3.5	108	30.7
	> 3.5	131	37.2
Residency	Urban	273	77.6
	Rural	79	22.4

### Technological and organizational characteristics

More than two-thirds of pharmacy students, 286 (81.3%), have access to one of the electronic devices, with the majority of respondents, 203 (57.7%), having access to smartphones. The findings show that approximately 207 (58.8%) of study participants did not receive basic computer training, and only half, 178 (50.6%) of the study participants, had sufficient skill to use computer systems, enabling them to use the Tele-pharmacy system. The majority of respondents, 298 (84.7%), said they had internet access in their learning environment, and roughly three-fourths, 210 (59.7%), said they used the internet to access health-related information.

Most of the 260 (73.9%) pharmacy students did not receive training in pharmacy information systems. Furthermore, more than two-thirds of 237 (67.3%) reported no pharmacy information system implementation guideline, and approximately 262 (74.4%) reported a lack of management support from their department to implement a Tele-pharmacy system Table 2.

**Table 2:** Technological and organizational characteristics of pharmacy students

Technological and organizational variables	Response	Frequency	Percentage
Access to electronic devices	Yes	286	81.3
	No	66	18.8
Which devices, did you have access to?	Smartphone	203	57.7
	Laptop	133	37.8
	Desktop	12	3.4
	Tablet computer	8	2.3
Have you ever taken basic computer training before?	Yes	145	41.2
	No	207	58.8
Self-reported basic computer skills	Sufficient	178	50.6
	Not sufficient	174	49.4
Access to the internet	Yes	298	84.7
	No	54	15.3
Mainly for what purpose do you use the internet	To get health information	210	59.7
	To communicate with my friends	103	29.3
	To get daily news	62	17.6
	To manage patients’ health data	18	5.1
	For reporting purpose	12	3.4
	Others	27	7.7
Have you ever taken any training related to pharmacy information systems?	Yes	92	26.1
	No	260	73.9
Availability of pharmacy information system implementation guideline	Yes	115	32.7
	No	237	67.3
Is there a management support to implement pharmacy information system from your department?	Yes	90	25.6
	No	262	74.4

### Pharmacy student’s knowledge regarding Tele-pharmacy

**Fig. 1:**
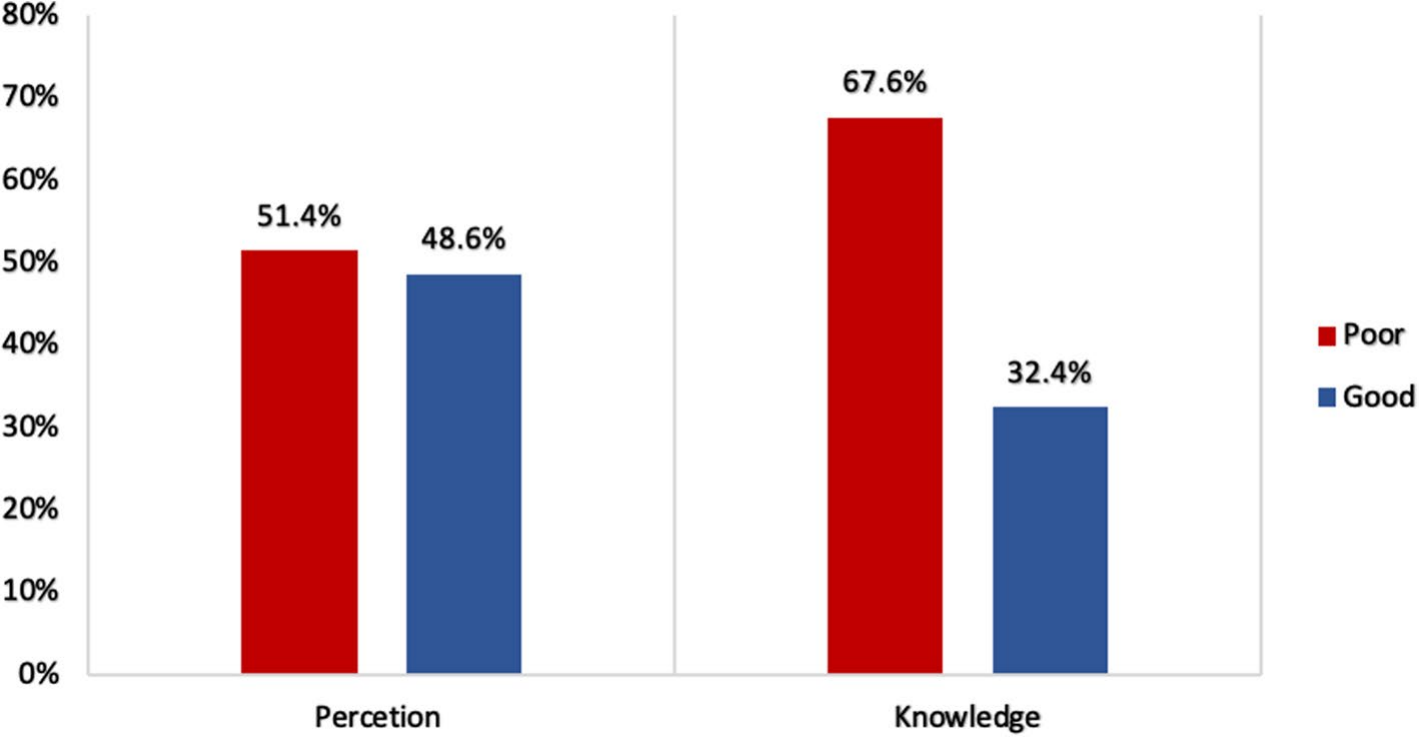
Pharmacy students’ knowledge and perception of Tele-pharmacy

Of the study participants, 114 (32.4% CI = 27%-37%) had adequate knowledge of the Tele-pharmacy system (Fig. 1. More than three-quarters, 304(86.4%) of the participants stated that they are unaware of Tele-pharmacy systems in Ethiopia. Approximately 277 (78.7%) of participants agreed that pharmacists should know about information and communication technology to practice Tele-pharmacy.

Approximately 80% of participants believed that tele-pharmacy played a significant role in outbreaks worldwide. The majority of 283 (80.4%) of the study participants agreed that Tele-pharmacy provides better counseling in terms of privacy and length of the session, Table 3.

**Table 3:** Knowledge of Tele-pharmacy among pharmacy students

Knowledge regarding Tele-pharmacy statements	Yes	No
Tele-pharmacy is available in Ethiopia	48(13.6)	304(86.4)
Information Communication Technology (ICT) knowledge is important for pharmacists on how to conduct Tele-pharmacy	277(78.7)	75(21.3)
Tele-pharmacy played a big role during outbreak around the world	280(79.5)	72(20.5)
Tele-pharmacy does require a strong internet connection or high-performance technology	245(69.6)	107(30.4)
Tele-pharmacy provides better counseling in terms of privacy and length of the session	283(80.4)	69(19.6)
Tele-pharmacy solves the waiting time problem in most general hospitals	283(80.4)	69(19.6)
Tele-pharmacy is also involved in ADR monitoring and reporting	238(67.6)	114(32.4)
In general hospitals, Tele-pharmacy is conducted by drug information service during office hours and by emergency departments after office hours	233(66.2)	119(33.8)
Patients from rural areas can have more medication access and information via Tele-pharmacy	168(47.7)	184(52.3)
Tele-pharmacy services can extend hospital pharmacy services outside office hours that do not offer round-the-clock pharmacy services	227(64.5)	125(35.5)

### Pharmacy student’s perception of Tele-pharmacy

Of the total study participants, 171 (48.6% CI = 43%—54%) had a positive perception of Tele-pharmacy Fig. 1. Table 4 illustrates that about 195(55.4%) agreed that Tele-pharmacy can help patients save their money and travel time to reach healthcare facilities. The majority of 245(69.6%) of the study participants agreed that Tele-pharmacy could minimize the cost of establishing a pharmaceutical business compared to a regular pharmacy. Moreover, about 216(61.4) of the study participants agreed that pharmacy schools should provide education programs on computers, IT, and Tele-pharmacy to assist in the future utilization of Tele-pharmacy Table 4.

**Table 4:** Perception towards Tele-pharmacy among pharmacy students

Perception towards Tele-pharmacy Questions	Strongly Disagree	Disagree	Neutral	Agree	Strongly agree
Do you think Tele-pharmacy will improve the patient’s adherence to the medication?	12(3.4)	24(6.8)	17(4.8)	224(63.6)	75(21.3)
Do you agree Tele-pharmacy will have a higher error rate for medication dispensing and filling, as compared to traditional pharmacy?	39(11.1)	53(15.1)	47(13.4)	177(50.3)	36(10.2)
Do you feel Tele-pharmacy will enhance the patient’s access to the medication, especially those who are in rural areas?	12(3.4)	61(17.3)	21(6.0)	189(53.7)	69(19.6)
Do you think Tele-pharmacy will provide the complete privacy setting during the consultation period?	9(2.6)	27(7.7)	51(14.5)	193(54.8)	72(20.5)
Based on your opinion, do you agree Tele-pharmacy will increase the pharmacist’s workload and commitment?	30(8.5)	76(21.6)	30(8.5)	162(46.0)	54(15.3)
Do you think Tele-pharmacy is able to help patients save their money and travel time to reach the healthcare facilities?	3(0.9)	17(4.8)	9(2.6)	195(55.4)	128(36.4)
Are you willing to share your personal information on the online database when using Tele-pharmacy services?	11(3.1)	21(6.0)	54(15.3)	191(54.3)	75(21.3)
Do you think Tele-pharmacy can minimize the cost to establish pharmaceutical business in comparison to regular pharmacy?	-	29(8.2)	24(6.8)	245(69.6)	54(15.3)
Do you think patient consultation via Tele-pharmacy will be effective?	6(1.7)	26(7.4)	33(9.4)	239(67.9)	48(13.6)
Do you think pharmacy schools should provide education programs on computers, IT, and Tele-pharmacy to assist in future utilization of Tele-pharmacy?	8(2.3)	9(2.6)	18(5.1)	216(61.4)	101(28.7)
Do you think therapeutic drug monitoring via Tele-pharmacy in rural areas will be easily monitored?	30(8.5)	72(20.5)	43(12.2)	141(40.1)	66(18.8)
Do you agree that security is a greater concern in a remote site Tele-pharmacy than in a traditional community pharmacy?	9(2.6)	56(15.9)	45(12.8)	173(49.1)	69(19.6)
Scarcity of pharmacists has caused a situation where medications are supplied without the involvement of pharmacists	18(5.1)	30(8.5)	54(15.3)	163(46.3)	87(24.7)
Do you agree that Tele-pharmacy is able to help to minimize this scarcity?	15(4.3)	6(1.7)	39(11.1)	211(59.9)	81(23.0)

### Factors associated with pharmacy students’ knowledge about Tele-pharmacy

Bivariate and multivariable analyses were used to investigate the factors associated with students' knowledge of Tele-pharmacy. In a bivariate analysis, the candidate variables for the multivariable logistic regression analysis were Sex, Age, Student’s grade (CPGA), Training related to pharmacy information systems, Device access, Basic computer training, computer skill, internet access, availability of pharmacy information system implementation guidelines, and management support to implement a pharmacy information system.

According to the findings of multivariable logistic regression analysis, being age group of 26–30 (AOR = 0.35, 95% CI: 0.17–0.68), being male (AOR = 2.38, 95% CI: 1.26–4.49), Having a CPGA of > 3.5 (AOR = 2.28, 95% CI: 1.24–4.19), Taking basic computer training (AOR = 2.00, 95% CI: 1.17–3.39), Management support to implement pharmacy information system (AOR = 1.84, 95% CI: 1.06–3.19) were found to be significantly associated with knowledge towards tele-pharamcy among pharmacy students Table 5.

**Table 5:** Factors associated with pharmacy students’ knowledge about Tele-pharmacy (*N* = 352)

Characteristics		Knowledge		COR (CI 95%)	AOR (CI 95%)
		**Good (%)**	**Poor (%)**		
Age	20–25	93(26.4)	166(47.2)	1	1
	26–30	15(4.3)	60(17.0)	0.44(0.24–0.83)	0.35(0.17–0.68) *
	> 31	6(1.7)	12(3.4)	0.89(0.32–2.45)	0.97(0.36–3.15)
Sex	Male	96(27.3)	178(50.6)	1.79(1.00–3.21)	2.38(1.26–4.49) *
	Female	18(5.1)	60(17.0)	1	1
CGPA	< 3.00	27(7.7)	86(24.4)	1	1
	3.0–3.5	36(10.2)	72(20.5)	1.59(0.88–2.87)	1.70(0.90–3.21)
	> 3.5	51(14.5)	80(22.7)	2.03(1.16–3.54)	2.28(1.24–4.19) *
Access to electronic devices	Yes	97(27.6)	189(53.7)	1.47(0.80–2.70)	1.36(0.70–2.63)
	No	17(4.8)	49(13.9)	1	1
Computer training	Yes	60(17.0)	85(24.1)	2.00(1.27–3.14)	2.00(1.17–3.39) *
	No	54(15.3)	153(43.5)	1	1
Computer skill	Yes	65(18.1)	113(32.1)	1.47(0.93–2.30)	1.27(0.74–2.17)
	No	49(13.9)	125(35.5)	1	1
Internet access	Yes	100(28.4)	198(56.3)	1.44(0.75–2.77)	1.23(0.60–2.51)
	No	14(4.0)	40(11.4)	1	1
Training related to pharmacy information systems	Yes	36(10.2)	56(15.9)	1.50(0.91–2.46)	1.01(0.56–1.82)
	No	78(22.2)	182(51.7)	1	1
Availability of pharmacy information system implementation guideline	Yes	44(12.5)	71(20.2)	1.47(0.92–2.36)	1.16(0.66–2.03)
	No	70(19.9)	167(47.4)	1	1
Management support to implement pharmacy information system	Yes	39(11.1)	51(14.5)	1.90(1.16–3.13)	1.84(1.06–3.19) *
	No	75(21.3)	187(53.1)	1	1

N:B *P-value < 0.05 for the multivariable analysis

### Factors associated with pharmacy students’ perception of Tele-pharmacy

Based on the multivariable logistic regression analysis in Table 5, Having access to electronic devices (AOR = 3.80, 95% CI: 1.81–7.97), training related to pharmacy information systems (AOR = 6.66, 95% CI: 3.34–13.29), availability of pharmacy information system implementation guideline (AOR = 2.99, 95% CI: 1.62–5.50) were found to be significantly associated with perception towards Tele-pharmacy among pharmacy students Table 6.

**Table 6:** Factors associated with pharmacy students’ perception of Tele-pharmacy (*N* = 352)

Characteristics		Perception		COR (CI 95%)	AOR (CI 95%)
		**Good (%)**	**Poor (%)**		
Sex	Male	130(36.9)	144(40.9)	0.81(0.49–1.34)	0.64(0.35–1.19)
	Female	41(11.6)	37(10.5)	1	1
Access to electronic devices	Yes	153(43.5)	133(37.8)	3.06(1.70–5.53)	3.80(1.81–7.97) *
	No	18(5.1)	48(13.6)	1	1
Computer training	Yes	95(27.0)	50(14.2)	3.27(2.10–5.10)	1.58(0.92–2.72)
	No	76(21.6)	131(37.2)	1	1
Computer skill	Yes	106(30.1)	72(20.5)	2.46(1.60–3.79)	1.23(0.72–2.08)
	No	65(18.5)	109(31.0)	1	1
Internet access	Yes	152(43.2)	146(41.5)	1.91(1.04–3.50)	1.87(0.92–3.83)
	No	19(5.4)	35(9.9)	1	1
Training related to pharmacy information systems	Yes	79(22.4)	13(3.7)	11.09(5.85–21.0)	6.66(3.34–13.29) *
	No	92(26.1)	168(47.7)	1	1
Availability of pharmacy information system implementation guideline	Yes	81(23.0)	34(9.7)	3.89(2.41–6.28)	2.99(1.62–5.50) *
	No	90(25.6)	147(41.8)	1	1
Management support to implement pharmacy information system	Yes	59(16.8)	31(8.8)	2.54(1.54–4.19)	1.62(0.86–3.01)
	No	112(31.8)	150(42.6)	1	1
Knowledge	Good	62(17.6)	52(14.8)	1.41(0.90–2.20)	1.09(0.63–1.88)
	Poor	109(31.0)	129(36.6)	1	1

N:B *P-value < 0.05 for the multivariable analysis

## Discussion

The finding of this study revealed that 32.4% (95% CI = 27%-37%) of pharmacy students had adequate knowledge regarding telepharmacy. The result of this study is lower as compared with studies conducted in Malaysia 67% [4], Saudi Arabia 42% [19], and the United States of America 60% [8]. The significant disparity could be attributed to the fact that developing countries use fewer eHealth applications than middle-income and developed countries [20, 21]. Because of the lower use of Tele-pharmacy in developing countries, such as Ethiopia, there is a significant knowledge gap between developed and developing countries. Another reason for the disparity could be country-specific differences in information and communication technology infrastructure and socioeconomic status [22].

In this study, 48.6% (95% CI = 43%—54%) of pharmacy students had a favorable perception of the Tele-pharmacy system. This finding is lower as compared with a study conducted in Malaysia with 61% [4], Saudi Arabia with 87% (11), Jordan 70.6% [10], and the United States 87% [8]. This could be due to technological advancement and ICT infrastructure differences between countries. Furthermore, the difference could be due to differences in educational curricula between countries; for example, in Ethiopia, pharmacy students only take fundamentals of health informatics courses. As a result, delivering an eHealth application course that includes Tele-pharmacy as part of their educational curriculum is highly recommended.

This research also found factors associated with pharmacy students' knowledge and perception of the Tele-pharmacy system.

Among the factors associated with knowledge, students in older age groups were 65% less likely to have sufficient knowledge of tele-pharmacy compared to students in younger age groups. This outcome is consistent with past research that showed younger students had a higher comprehension of health information technologies [23–25]. This was explained by the fact that most university students in the Ethiopian were under the age of 25, and younger students were more active in using and accessing information and communication technology [26].

This study found that male respondents were 2.38 times more likely to have adequate knowledge about Tele-pharmacy than females. Other research has also found that men understand eHealth applications better than women [27–29]. The digital divide could explain the difference and gender inequality in access to technology continue to be challenging in low-income countries like Ethiopia. This implied that female students would receive more attention to improve their understanding of eHealth applications. Moreover, students Having a CPGA of > 3.5 were 2.28 times more likely to have adequate knowledge of the Tele-pharmacy system. This was explained by the fact that students with higher CPGA were more likely to understand the Tele-pharmacy system than students with lower CPGA.

Pharmacy students who took training on a basic computer were 2.00 times more likely to have adequate knowledge of Tele-pharmacy than students who did not receive basic computer training. This study's findings are consistent with previous research indicating that computer training can improve understanding of the Telehealth applications [30–32]. This is because basic computer training is the most critical factor in improving students' knowledge of Tele-pharmacy [33]. This implies that computer training is an integral part of successful Tele-pharmacy adoption. Furthermore, those with management support were 1.84 times more likely to have good knowledge of Tele-pharmacy than those who did not have management support. This implies that health administrators should pay attention and provide ongoing support to future pharmacists to understand better and implement a Tele-pharmacy system.

Among the factors significantly associated with pharmacy students' perceptions of Tele-pharmacy, having access to electronic devices was 3.80 times more likely to have a positive perception than not having access to electronic devices. Previous research backs up this evidence that having access to electronic devices is the most critical factor in having a positive perception of the Telemedicine system [34, 35]. This implied that access to electronic devices was required to implement Ethiopia's Tele-pharmacy system successfully.

Those who received pharmacy information system training were 6.66 times more likely to have a positive perception of the Tele-pharmacy system than those who did not receive pharmacy technology training. This is because students who have received training in pharmacy technology may have a favorable opinion of the Tele-pharmacy system [10, 36]. Furthermore, the availability of pharmacy information system implementation guidelines was linked to a positive perception of the Tele-pharmacy system. Those with guidelines on pharmacy information system implementation were 2.99 times more likely to have a positive perception of Tele-pharmacy than their counterparts. This implies that health managers should prepare a user manual when planning to implement Telehealth applications in the Ethiopian healthcare system.

## Conclusion and recomendation

This study found that pharmacy students have limited knowledge and perceptions of the Tele-pharmacy system. A continuing Tele-pharmacy training package, incorporating pharmacy information system guidelines as part of their education, and providing managerial support could be recommended to improve pharmacy students' knowledge and perception of Tele-pharmacy. Based on the findings, policymakers and other stakeholders can develop a plan to implement Tele-pharmacy in the health care system.

### Strength and limitations of the study

This study is unique in Ethiopia because it discovered future pharmacists' knowledge and perceptions of Tele-pharmacy. However, the study has some limitations. The study's findings could be influenced by response bias. However, we attempted to reduce this bias by improving survey designs through pre-testing feedback.

## Data Availability

This article includes all the data generated and analyzed during this study.

## Abbreviations

AOR: Adjusted odds ratio
CI: Confidence Interval
COR: Crude odds ratio
ADR: Adverse drug reaction
e-Health: Electronic health
ICT: Information communication technology
SPSS: Statistical package for social science
